# Exploring cell death mechanisms in spheroid cultures using a novel application of the RIP3-caspase3-assay

**DOI:** 10.1038/s41598-024-66805-4

**Published:** 2024-07-11

**Authors:** C. I. Philippi, J. Hagens, K. M. Heuer, H. C. Schmidt, P. Schuppert, L. Pagerols Raluy, M. Trochimiuk, Z. Li, M. J. Bunders, K. Reinshagen, C. Tomuschat

**Affiliations:** 1 Department of Pediatric Surgery, University Medical Center Hamburg-Eppendorf, Hamburg, Germany; 2 Research Department of Virus Immunology, Leibniz Institute of Virology, Hamburg, Germany; 3 Division of Regenerative Medicine and Immunology, III. Department of Medicine, University Medical Center Hamburg-Eppendorf, Hamburg, Germany

**Keywords:** Organoids, Cell death mechanisms, RIP3-caspase3 assay, TNFα -induced stress, Apoptosis and necroptosis, Paediatric research, Stem-cell research, Cell death and immune response, Intestinal stem cells

## Abstract

This study explores the application of the RIP3-caspase3-assay in heterogeneous spheroid cultures to analyze cell death pathways, emphasizing the nuanced roles of apoptosis and necroptosis. By employing directly conjugated monoclonal antibodies, we provide detailed insights into the complex mechanisms of cell death. Our findings demonstrate the assay’s capability to differentiate between RIP1-independent apoptosis, necroptosis, and RIP1-dependent apoptosis, marking a significant advancement in organoid research. Additionally, we investigate the effects of TNFα on isolated intestinal epithelial cells, revealing a concentration-dependent response and an adaptive or threshold reaction to TNFα-induced stress. The results indicate a preference for RIP1-independent cell death pathways upon TNFα stimulation, with a notable increase in apoptosis and a secondary role of necroptosis. Our research underscores the importance of the RIP3-caspase3-assay in understanding cell death mechanisms in organoid cultures, offering valuable insights for disease modeling and the development of targeted therapies. The assay’s adaptability and robustness in spheroid cultures enhances its potential as a tool in personalized medicine and translational research.

## Introduction

The intricate processes of cell death, including apoptosis and necroptosis, are fundamental to our understanding of various pathological conditions. In particular, the balance between these processes is crucial in conditions of inflammation, neurodegenerative diseases, or cancer^1–3^. Despite advancements in cellular biology, the accurate delineation of these processes remains a significant challenge, especially in physiologically relevant models like organoids. Recent advances in organoid technology offer promising avenues for more accurate disease modeling, providing three-dimensional structures that closely mimic in vivo conditions^4^. However, applying existing cell death assays to these models is challenged by their cellular and structural complexity. Traditional methods often fall short in providing a detailed analysis of these complex pathways, especially in heterogeneous cell populations like those found in organoid cultures. For determination of cell death, fluorescence microscopy using specific apoptosis indicators like annexin V, propidium iodide (PI) or the TUNEL assay have been described in three-dimensional cell cultures^5–8^. The annexin V analysis is a superior approach, not only allowing the quantification of apoptosis but also differentiating between early and late stages of apoptosis^5^. However, the distinction between apoptosis and necroptosis is impossible, as both conditions can lead to externalization of phosphatidylserine. A combined immunostaining of apoptosis indicators like cleaved-caspase 3 or PI with additional markers is a common method to visualize the cells viability and apoptotic status^6,7^. Immunofluorescence staining may be complemented with live cell imaging, which has often been promoted for three-dimensional cell cultures. But notably, phototoxic effects during the analysis can limit cell vitality and thus lead to false results^9^. As proposed by Firestein et al. (2021), DNA fragmentation can be detected using a TUNEL assay, but could not stand the application onto larger sample cohorts, limiting the value for enteroid research^7,10^. Apart from imaging methods, techniques using enzymatic detection such as the M30 assay or measurements with mitochondrial enzymes (MTT assay) have been applicated to determine cell viability in enteroids, however, both assays are highly cell type dependent and lack the ability to distinguish between different forms of cell death^7,11^.

The limitations described hinder our ability to fully understand and effectively target dysregulated cell death mechanisms in disease settings^12,13^. There is a critical need for assays that can differentiate between various cell death pathways in a single, cohesive analysis. Addressing these challenges is crucial for the development of targeted therapies and enhancing our understanding of disease mechanisms at a cellular level^14,15^.

In this context, our study introduces the RIP3-caspase3-assay, a novel approach employing directly conjugated monoclonal antibodies to analyze regulated cell death mechanisms in spheroid cultures. This assay is designed to overcome the limitations of existing methods, offering a detailed and nuanced analysis of cell death pathways in heterogeneous organoid samples. By providing insights into RIP1-independent apoptosis, necroptosis, and RIP1-dependent apoptosis, the RIP3-caspase3-assay aims to advance our understanding of cell death in disease processes and aid in the development of more effective therapeutic strategies^16^.

## Material and methods

### Cultivation of organoids

The study was conducted according to the guidelines of the Declaration of Helsinki and approved by the Institutional Review Board Hamburg ethics committee. Parents or guardians provided informed consent for collection and analyses of the tissues. Tissue samples were harvested from patients who underwent surgery for Morbus Hirschsprung pull-through procedure (n = 3), intestinal atresia repair (n = 1) or colostomy closure (n = 1) (patient characteristics see Table 2s, supplementary material). Samples were transported in Iscove’s Modified Dulbecco’s medium (IMDM, #12440053, Gibco ThermoFisher Scientific, Waltham, MA, USA) containing 20% fetal bovine serum (FBS, #0500-064, ThermoFisher Scientific, Waltham, MA, USA) and 1% penicillin/streptomycin (P/S, #PS/B, Capricorn Scientific, Ebsdorfergrund, Germany) for viability preservation and processed within 24 h. Samples were washed in sterile Dulbecco’s phosphate-buffered saline (DPBS; #37350, Gibco Thermo Fisher Scientific, USA). The colonic mucosa was mechanically separated from the rest of the tissue and sliced into pieces measuring 1–2 mm. The pieces were incubated in IMDM containing 5 mM ethylenediaminetetraacetic acid (#15575-038, Invitrogen, Waltham, MA, USA) and 2 mM DL-Dithiothreitol (DTT, #D9779-5G, Sigma–Aldrich, St. Louis, MO, USA) for 20 min at 4 °C. Crypt isolation was verified using an inverse microscope (Olympus IX50-S8F; Olympus, Tokyo, Japan). The medium containing the mucosa was pipetted up and down several times with a 25 ml serologic pipette, followed by another incubation at 4 °C for 20 min. Adult stem cells (AdSCs) were isolated using a 70 μm cell strainer (#352350, Corning, Corning, NY, USA) and rinsed with a washing buffer containing IMDM with 2% FBS and 1% P/S to collect all cells. The cells were centrifuged at 500×*g* at 4 °C for 10 min and washed twice with Advanced Dulbecco’s Modified Eagle Medium (Advanced DMEM, #12491-015, Gibco ThermoFisher Scientific, USA) containing 1% 4-(2-hydroxyethyl)-1-piperazineethanesulfonic acid (HEPES, #H3537-100ML, Sigma–Aldrich, St. Louis, MO, USA), 1% GlutaMAX (#35050-061, Gibco ThermoFisher Scientific, Waltham, MA, USA), and 1% P/S (further referred to as Adv. +++). 20 µl cell suspension was pipetted into 40 μl of growth factor-reduced, phenol red-free Matrigel matrix (#356231, Corning, Corning, NY, USA) and mixed carefully. 30 μl of the mixture was pipetted into a prewarmed flat bottom 24-well-plate (#3526, Costar, Corning, NY, USA). Domes solidified upon incubation at 37 °C and 5.0% CO_2_ for 30 min. 500 µL of seeding medium consisting of IntestiCult organoid growth medium human (OGM-h, #060610, Stemcell Technologies, Canada) supplemented with 5 mM of ROCK-pathway inhibitor (ROC, #72302, Stemcell Technologies, Vancouver, Canada), 1% P/S, and 0.02% Primocin (#ant-pm-05, InvivoGen, San Diego, CA, USA) was added to the wells. Successful seeding was confirmed by light microscopy. Organoids were cultivated at 37 °C and 5.0% CO_2_. The seeding medium was changed every 2–3 days. After 7–10 days, organoids were ready for the first passage.

### Maintenance of the organoids

Passaging of organoids was performed as follows. The medium was removed and the Matrigel domes were broken up by pipetting with 1 ml Adv. +++ . The wells were washed with Adv. +++ and cell suspension was centrifuged at 300×*g* and 4 °C for 5 min. Organoid cells were separated into smaller cell clusters by pipetting up and down several times, and again centrifuged at 400×*g*. After removing the supernatant, disrupted organoids were resuspended with Adv. +  +  + and reseeded in Matrigel. Medium was changed every two to three days.

After the fifth passage, organoids were considered mature and used for the experiments. After 4–6 days, OGM-h was replaced with IntestiCult organoid differentiation medium human (ODM-h, #100-02114, StemCell Technologies, Vancouver, Canada) containing 5 mM daptomycin (DAPT), a notch pathway inhibitor (#72080, StemCell Technologies, Vancouver, Canada). Organoids were cultured for 3–5 days in ODM-h, which was changed every 2 days as well. Once the epithelia of the organoids started to thicken and form bud-like structures, organoids were prepared for the experiment. Organoid growth and differentiation were monitored using light microscopy (Leica DM IL LED, Leica Microsystems, Wetzlar, Germany).

### Determination of TNFα concentration using flow cytometry

**Figure 1:**
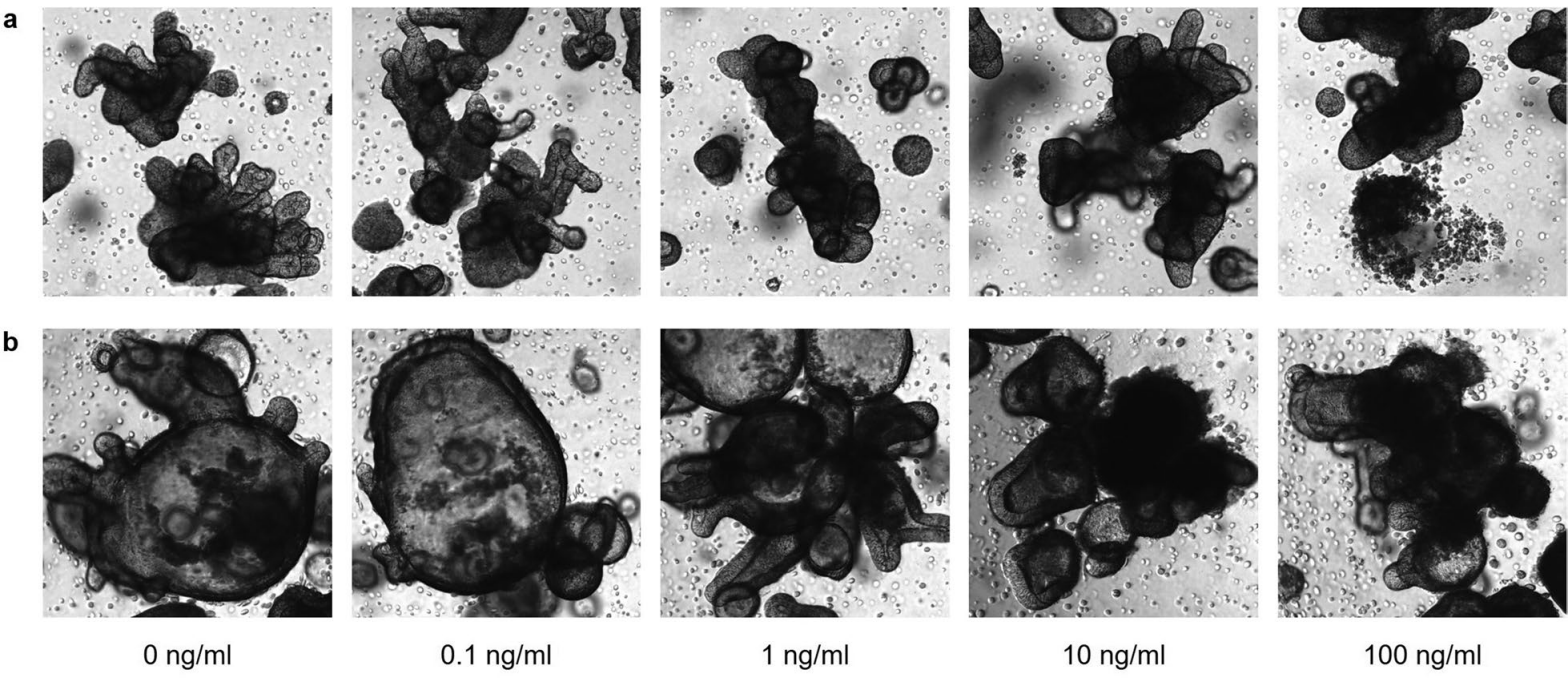
Light microscopy imaging (magnification ×4) of two exemplary organoid cultures (**a**,**b**) after stimulation with increasing TNFα concentrations (0, 0.1, 1, 10 and 100 ng/ml). Images were taken immediately before processing for flow cytometry after 72 h of proinflammatory stimulation. Without cytokine influence, enteroids present complex budding structures without accelerative debris. With increasing concentrations of TNFα, the enteroids present visible morphological changes with rounded buds and cell detritus inside the lumen as a sign for cell destruction, especially following concentrations 10 ng/ml and 100 ng/ml.

The purpose of this experiment was to assess the effect of tumor necrosis factor-α (TNFα) on differentiated organoids. Ten wells containing 30 μl Matrigel dome were needed from each culture of organoids to perform the experiment. Organoids were maintained for 5 days in OGM-h. On day 5, the medium was replaced with supplemented ODM-h to initiate differentiation. After 24 h, TNFα (TNFα, #300-01A, PeproTech London, UK) was added to the ODM-h in the concentrations 0,1 ng/ml, 1 ng/ml, 10 ng/ml, 100 ng/ml, and an unstimulated control, always treating two wells the same. Organoids were maintained in ODM-h with TNFα for a total of 72 h, with one medium change in between (Fig. 3s, supplementary material). Organoid growth was monitored using light microscopy (Fig. 1).

For flow cytometry analysis of the cells, the medium was removed and Matrigel containing the organoids was pipetted up and down with Adv. +++ , transferred into a 15 ml tube (#188271, Greiner Bio-One, Kremsmünster, Austria), and incubated with TrypLE (#12605028, Gibco ThermoFisher Scientific, Waltham, MA, USA) at 37 °C for 4 min to separate the organoids into smaller cell clusters. The organoid fragments were further separated into single cells by pipetting. Cell isolation was verified using a light microscope. The cells were centrifuged in Adv. +++ at 300×*g* and 4 °C for 5 min and the supernatant was discarded. The cells were incubated with Zombie NIR fluorescent dye (#423105, dilution 1:2000, BioLegend, San Diego, CA, USA) for 30 min at 4 °C in the dark. Next, the cells were washed and fixed with an intracellular fixation buffer (eBioFix, #00–8222-49, BD Biosciences, San Jose, CA, USA) for 20 min at 4 °C. Cells were resuspended in 200 μl DPBS and ready for analysis with LSR Fortessa and BD FACS Diva Software (version 8.0.2, firmware version 1.13, BD Biosciences, San Jose, CA, USA). Zombie NIR was excited by a 633 nm laser and collected in a 780/60 nm detector. Compensation was not necessary using only one laser for this analysis. Gating was set using unstained samples. For each tube, 10,000 single cells were analyzed. Small debris at the origin was removed using a gate in the FSC-A vs. SSC-A dot plot. Single cells were gated on SSC-W versus SSC-H dot plots. These were gated on a dot plot of Zombie NIR vs. SSC-A showing all zombie-positive dyed cells. Zombie staining indicated a compromised cell membrane, these cells were defined as dead.

### RIP3-caspase3-analysis of apoptosis, necroptosis and RIP1-dependent apoptosis

To quantify RIP1-independent apoptosis, RIP1-dependent apoptosis, necroptosis, and other forms of cell death in the epithelial cells, the intracellular cell-death proteins RIP3 and caspase3 were stained in addition to Zombie NIR and measured by flow cytometry, as described by Lee et al. (2018)^16^. Sensitivity to the stressor was measured by the extent of cell death for each sample and condition. Untreated samples served as negative controls.

For this experiment, four wells containing a 30 μl Matrigel dome were used from each culture. The organoids were maintained in OGM-h for 5 days. On day 5, the medium was replaced with ODM-h. 48 h before flow cytometry, two of the four wells were treated with TNFα at a concentration of 100 ng/ml. The other two wells were maintained in ODM-h without TNFα (Fig. 4s, supplementary material).

Organoids were prepared for flow cytometry as described in Sect “Determination of TNFα concentration using flow cytometry”. After the fixation with eBioFix, cells were permeabilized with PBS containing 0.25% Triton X-100 (#T8787-50ML, Sigma–Aldrich, St. Louis, MO, USA) for 15 min at 4 °C under movement to expose intracellular targets. The cells were incubated with fluorescent-conjugated antibodies anti-active caspase-3-BV650 (#564096, dilution 1:50; BD Biosciences, San Jose, CA, USA) and anti-RIP3-Alexa Fluor 488 (clone B-2, #sc-374639, dilution 1:125, Santa Cruz Biotechnology, Dallas, TX, USA) for 30 min at 4 °C in the dark. Cells were washed with 500 µl PBS containing 0.25% Triton X-100 and then resuspended in 200 µl PBS. In each tube, 5000 single cells were analyzed using LSR Fortessa and BD FACS Diva Software. Zombie NIR was excited by a 633 nm laser and collected in a 780/60 nm detector. Caspase-3-BV650 was excited by a 405 nm laser and collected at 675/30 nm. RIP3-Alexa Flour 488 was excited by a 488 nm laser and collected at 585/40 nm. The lasers were compensated with unstained samples using the BD FACS Diva Software. Gates were set by using fluorescence minus one controls as a standard procedure. Small debris was removed using a gate in the FSC-A vs. SSC-A dot plot. Single cells were gated on SSC-W versus SSC-H dot plots. These were gated on a dot plot of Zombie NIR vs. SSC-A showing all zombie-positive dyed cells that were defined as dead. Cells unstained by Zombie NIR were gated in the FSC-A vs. SSC-A dot plot and were defined to be living cells. Using caspase-3-BV650 vs. RIP3-Alexa Fluor 488 dot plots, live and dead cells were gated separately with the same gate configuration for each plot. Caspase3-positive cells indicate a RIP1-independent apoptotic process, and RIP3-positive cells indicated a necroptotic process. Caspase3- and RIP3-positive cells were determined to undergo RIP1-dependent apoptosis. Cells with neither caspase3 nor RIP3 signals were assumed to have undergone other forms of cell death (Table 1).

**Table 1:** Cell death status definitions.

	RIP3-status	Caspase3-status
RIP1-dependent Apoptosis	Positive	Positive
Apoptosis	Negative	Positive
Necroptosis	Positive	Negative
Other cell death	Negative	Negative

Caspase3-positive signal indicating an apoptotic cell, RIP3-positive signal indicating a necroptotic cell, caspase3- and RIP3-positive signal indicating a RIP1-dependent apoptosis and no caspase3- or RIP3-signal indicating other forms of cell death.

### Statistical analysis

Statistical analyses were generated using GraphPad Prism 9 (San Diego, CA, USA). Simple comparisons were performed by descriptive statistics and student’s *t*-test. Mean values of multiple variables were compared using two-way analysis of variance (ANOVA). *p*-values < 0.05 were considered statistically significant.

## Results

### Determination of TNFα concentration

This experiment was performed to determine the effect of increasing TNFα concentrations on overall cell survival, using Zombie NIR staining only. Stimulation with TNFα showed stable percentages of Zombie^+^ cells between 0 ng/ml and 10 ng/ml, ranging from 39.06% (± 8.51%) to 42.68% (± 7.35%). Cell death rate increased to 45.30% (± 7.05%) after treatment with 100 ng/ml.

The FSC-A vs. SSC-A dot plot detected an increase in cell debris after 72 h treatment with TNFα as a display of organoid damage. Consequently, only 5000 single cells were analyzed in each tube, ensuring comparability between the different treatments and samples. The destruction was already visible at 48 h of treatment during daily monitoring (Fig. 1). Therefore, for the final next experimental analysis setup, 100 ng/ml TNFα for 48 h was considered the optimal condition to induce stress in the cells.

### Cell death markers RIP3, caspase3 and Zombie can be detected in organoids using the RIP3-caspase3-Assay

**Figure 2:**
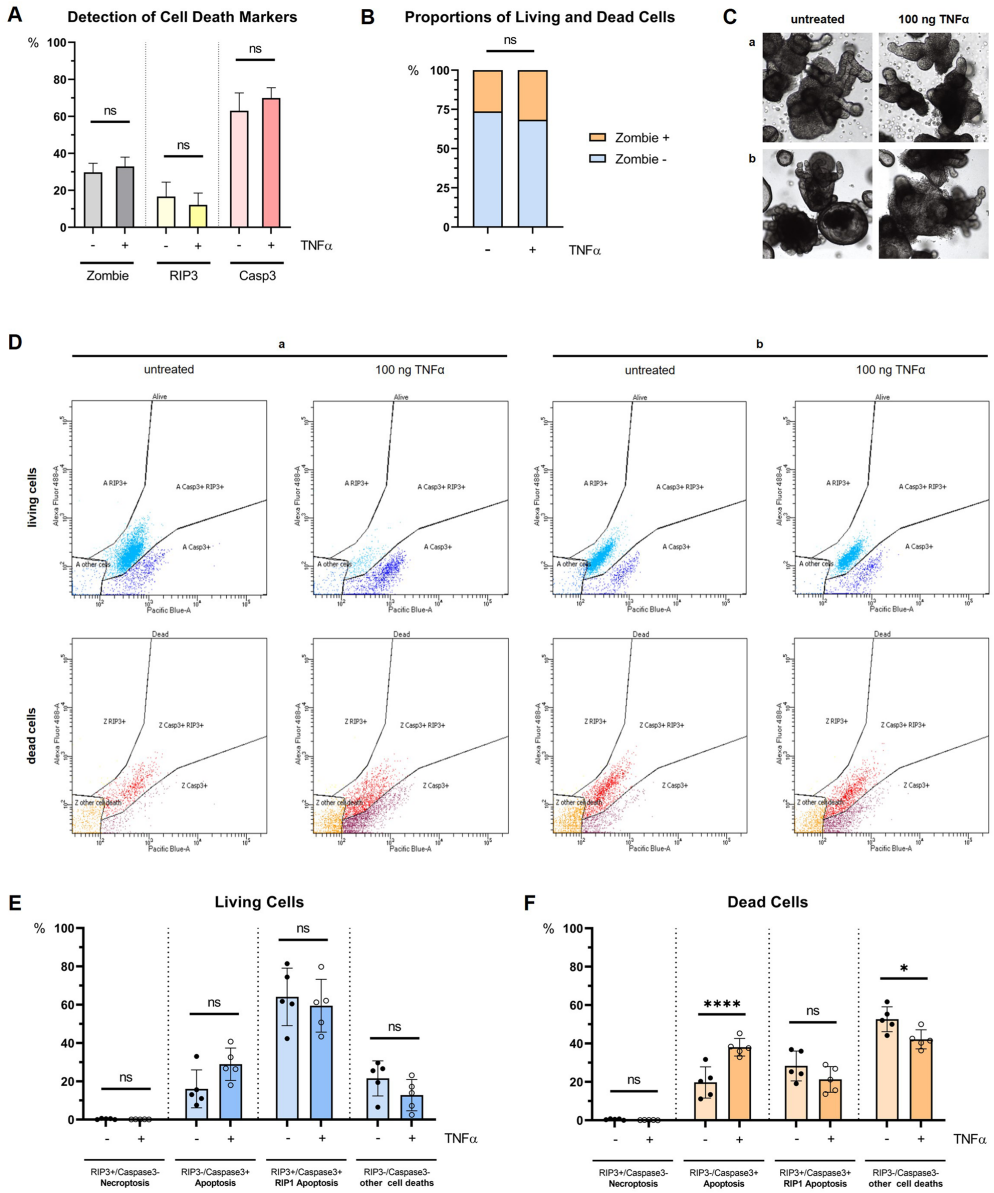
Summary of experimental findings using the RIP3-caspase3-assay. (**A**) Measurement of assay markers Zombie NIR, caspase3 and RIP3. (**B**) Proportions of Zombie+ (dead) cells and Zombie- (living) cells before ( −) and after treatment ( +) with 100 ng/ml TNFα. Treatment results in an increase in dead cells. (**C**) Light microscopy image of organoids from two representative patients (a, b) before (left) and after stimulation (right) with 100 ng/ml TNFα. Visible organoid impairment after treatment with TNFα for 48 h. (**D**) FACS dot plots for two exemplary samples (a, b) for living (upper row, blue) and dead (lower row, red) cells with and without TNFα treatment. Using caspase-3-BV650 (Pacific Blue-A) versus RIP3-Alexa Fluor 488 dot plots, live and dead cells were gated separately with the same gate configuration for each plot. RIP3^−^/caspase3^+^ cells indicate a RIP1-independent apoptotic process, and RIP3^+^/caspase3^−^ cells indicated a necroptotic process. Caspase3^+^/RIP3^+^ cells were determined to undergo RIP1-dependent apoptosis. Cells with RIP3^−^/caspase3^−^ signals were assumed to have undergone other forms of cell death. The dot plots visualize the overall shrinkage of cell amount after the treatment with TNFα as well as a shift from the RIP1-dependent apoptotic cells (RIP3^+^/caspase3^+^) to the non-dependent apoptotic cells (RIP3^−^/caspase3^+^). (**E**) Living cells undergoing cell death at measurement timepoint were defined by a Zombie-negative status. RIP1-dependent apoptosis represents the predominant cell death type. (**F**) Dead cells were defined by a Zombie-positive status. Other cell death types represent the largest group of dead cells. However, analysis reveals a significant shift to RIP1-independent apoptosis after treatment (*p* < 0.0001) with a loss in other forms of cell death (*p* = 0.0255).

As exemplary seen in Fig. 2C, stimulation with TNFα caused morphological changes indicating destruction within the cultures. As shown in Fig. 2A, the assay was able to detect Zombie staining as well as RIP3- and caspase3-status. Treatment with TNFα caused a slight increase in Zombie-cells (29.71% vs. 32.89%, *p* = 0.3451) and caspase3 expression (63.00% vs. 69.91%, *p* = 0.2038) while RIP3 was detected less after stimulation (16.61% vs. 12.13%, *p* = 0.3482). Proportions shifted towards a higher number of dead cells and away from living cells following TNFα treatment, as shown in Fig. 2B (*p* = 0.3380).

### RIP3-caspase3-assay reveals apoptosis as the leading cell death mechanism in living cells

For further analysis, cell death measurements were separated into living (Zombie^−^) and dead cells (Zombie^+^). Living cells represent cells with intact cell membranes not stained by Zombie NIR and were gated as described in Sect “RIP3-caspase3-analysis of apoptosis, necroptosis and RIP1-dependent apoptosis”. As seen in Fig. 2D–F, apoptosis was the leading cause of cell death. Comparing cell death mechanisms, predominance of RIP1-dependent apoptosis reached significance against all other groups pre and post treatment (*p* < 0.0001). Also, RIP1-independent apoptosis was significantly higher compared to necroptosis after stimulation (*p* = 0.0002), but not before (*p* = 0.0644). Comparison between RIP1-independent apoptosis and other forms of cell death also did not reach significance neither pre (*p* = 0.8027) nor post treatment (*p* = 0.0559) with TNFα.

Stimulation caused an increase in RIP3^−^/caspase3^+^ cells from 16.06 to 28.91% (*p* = 0.1572), while RIP3^+^/caspase3^+^ cells showed less change (64.08% vs. 59.47%, *p* = 0.9101). There were near to no detectable RIP3^+^/caspase3^−^ cells (0.33% vs. 0.13%, *p* > 0.9999). RIP3^−^/caspase3^−^ cells decreased from 21.54 to 12.79% (*p* = 0.4987) after the treatment.

### Dead cells show a significant rate of RIP1-independent apoptosis after TNFα stimulation

Zombie-positive cells, representing cells with compromised membranes, were also gated as described in Sect “RIP3-caspase3-analysis of apoptosis, necroptosis and RIP1-dependent apoptosis” and are displayed in Fig. 2D, F. Comparison between the different cell death mechanisms groups before and after treatment revealed significant differences (*p* < 0.0001) between all except three combinations: Before stimulation, RIP1-independent and RIP1-dependent apoptosis did not differ (*p* = 0.1307), but difference reached significance after TNFα treatment (*p* = 0.0003), showing a growth of the first and decrease of the latter one. Moreover, RIP1-independent apoptosis nearly reached the level of other cell deaths after stimulation (*p* = 0.8368).

While apoptosis overall remained a prominent cause of cell death, RIP3^−^/caspase3^+^ cells increased significantly from 19.72 to 38.05% (*p* < 0.0001) while RIP3^+^/caspase3^+^ cells decreased slightly from 28.31 to 21.31% (*p* = 0.2212). RIP3^+^/caspase3^−^ cells represented the smallest group with 0.47% before versus 0.13% (*p* > 0.9999) after treatment. RIP3^−^/caspase3^−^ cells presented the leading cause of cell degradation in this cohort, but also significantly decreased after treatment with TNFα (52.66% vs. 42.17%, *p* = 0.0255).

## Discussion

Our study’s successful application of the RIP3-caspase3-assay in heterogeneous organoids highlights its methodological strengths. By employing directly conjugated monoclonal antibodies, our assay provides a nuanced analysis of cell death pathways, revealing the complexity of these processes in greater detail. This approach aligns with Lee et al.’s findings, which distinguished between apoptosis, necroptosis, and RIP1-dependent apoptosis in both viable and non-viable cell populations^16^. Our extension of these advancements to spheroid cultures marks a first in the field, showcasing the assay’s reliability and its capability to discern between cell death mechanisms. In our study, the treatment of isolated intestinal epithelial cells from spheroids with TNFα accelerated the cell death rate, as indicated by Zombie-stained cells. The study observed an initial lower response to TNFα that increased with higher concentrations. In this context, this pattern suggests a possible adaptive or threshold response of the cells to TNFα-induced stress. TNFα is known to activate multiple signaling pathways, including NF-κB, MAPKs, and the apoptotic pathway^17^. The activation of NF-κB, for instance, can lead to the expression of survival genes, which might help cells adapt and survive under increasing TNFα concentrations^18^. However, in this study we had a linear response to the TNFα concentration with relevant damage of the organoids at 100 ng/ml after 48 h. This finding was in line with observed morphological changes of treated organoids (Fig. 1).

Upon TNFα stimulation, we observed an increase in caspase3 expression and a decrease in RIP3 expression across all samples. FACS analysis further revealed that TNFα-treated cells showed a significant increase in apoptosis, while simultaneously shifting away from RIP1-dependent apoptotic mechanisms, without involving necroptosis (Fig. 2D–F). This finding suggests a preference for RIP1-independent cell death in both living cells undergoing cell death and those that had already died. The shift towards apoptosis in the presence of TNFα is a key finding. Apoptosis is a programmed and orderly cell death process, often considered immunologically silent, while necroptosis is a form of programmed necrosis that is inflammatory in nature. The decision between apoptosis and necroptosis is often determined by the availability and activity of key regulatory proteins like RIP1, RIP3, and caspase8^18^. This shift in the type of cell death can also be attributed to the inflammatory context, where the co-sensing of multiple cytokines and the activation of various pathways, such as the non-canonical NF-κB pathway, lead to different forms of cell death. For instance, the combination of TNFα with other cytokines can result in TNFR1-induced RIP1 kinase activity-dependent apoptosis^18^. Also, the balance between apoptosis and necroptosis is depended on the concentration of TNFα^19^. In contrast, TNFα treatment did not impact the proportion of “other” cell death types substantially. However, cells sorted into this category showed a higher proportion in the dead cell section than in the living cells, which may be attributable to the disruption of cell membrane integrity and subsequent cell death during assay preparation.

Another important finding is the differential response in living versus dead cells (Fig. 2E, F). The significant changes in the dead cell population, as opposed to the living cells, suggest that TNFα effects are more pronounced in cells already compromised or stressed. This could imply that TNFα is a more potent inducer of cell death pathways in cells that are already damaged. This is because stressed or damaged cells might have compromised defense mechanisms (for example reduced expression of anti-apoptotic proteins), making them more prone to TNFα cytotoxic effects^18^. Also, the absence of necroptotic cells in both living and dead cells suggests that, under these conditions, TNFα does not induce necroptosis and underscores TNFα role in promoting apoptosis under these experimental conditions in colonic epithelial organoids.

A variety of cell death determination methods have been published for cell culture in general as well as organoids in particular. However, none of them were able to distinguish between RIP1-dependent and -independent apoptosis or necroptosis without profound extensions, since these methods label cell death status according to the presence or absence of either membrane or nucleus destruction without describing the underlying mechanism further^5–8^. Applying these methods on organoids, separation into single cells may often be required. Since this process is regularly performed through mechanic disruption, impairment of membrane integrity and technically induced cell death must be discussed as negative influencers of the results. Moreover, artifacts not only rely on structural, but also technical circumstances, such as the presence of basement membrane or special maintenance conditions. The RIP3-caspase3 assay’s ability to detect both major and minor cell populations undergoing various forms of cell death therefore is particularly valuable in the context of spheroid cultures, where cell heterogeneity and complexity are inherent^14^. Notably, unlike the original technique described by Lee et al., our approach did not use CalTag fixative, known to upregulate RIP3. This modification is noteworthy because the detection of RIP3 in our results is not influenced by the fixative. Since we were still able to detect RIP3 as an individual cell death marker in all organoids, suggests that RIP3 is inherently present in the organoids and is not an artifact of the fixation process. Interestingly, necroptotic RIP3^+^/Caspase3^−^ cells were not identified in downstream analysis, regardless of stimulation or Zombie status. Since RIP3 was present in RIP1-dependent apoptosis, as indicated by RIP3^+^/Caspase3^+^ status, the reasons why the assay did not determine necroptosis remain unclear. This could be due to several factors, such as the specific cellular context, the sensitivity of the assay, or other regulatory mechanisms in the cells that may inhibit necroptosis. To further explore this, our ongoing investigations are utilizing a cytokine cocktail containing IL-6 and IL-1ꞵ to simulate intestinal inflammation in a more holistic manner (Fig. 5s, supplementary material).

This study, while showcasing several strengths, also acknowledges certain limitations. Colonic organoid cultures, composed of different cell types like the colonic epithelium, provide insights into the composition and interaction of diverse cell populations in organoids. However, our analysis did not include cell type determination, which may mask specific dysregulations in signaling pathways. Additionally, for FACS analysis, organoids must be dissociated into single cells, a process that can inevitably cause cell damage and produce debris. This may increase the proportion of dead cells via two subsequent mechanisms: (1) Cells labeled double-negative for RIP3 and Caspase3 represent cells with relevant membrane impairment, as indicated by Zombie-positive staining, but still preserve enough cellular integrity to be registered as a single cell in flow cytometry; (2) In case of a complete cell disruption, fragments of the membrane and intracellular structures can be detected through Zombie staining. To mitigate these challenges, we employed lower centrifugal accelerations and carefully separated cell clusters using a dissociation reagent, which positively influenced cell death rates. Moreover, cell damage and death, accompanied by an increase in debris, were noted after differentiation periods exceeding 96 h. To ensure sufficient differentiation of the organoids while minimizing these effects, we precisely matched the supplement addition and time points of differentiation. Debris caused by total membrane disruption was excluded from further analysis through the gating strategy. Since there was limited time for the organoid cells to enter a coordinated cell death pathway before fixation was performed, artificial signals for either RIP3 or caspase3 based on mechanic influences were unlikely. Considering the varied growth rates of organoid cultures derived from primary patient material, ensuring comparability of our results required measuring the same number of single cells in each tube, leading to a smaller number of analyzed cells. The challenges in reaching the necessary cell count during assay preparation led to the exclusion of samples with lower cell numbers, with minimal cell loss achieved by transferring organoids twice during preparation. Despite these challenges, the robustness of our results across a highly heterogeneous set of organoids highlights the substantial broader implications of this methodological innovation. The assay’s adaptability to spheroid cultures significantly enhances our understanding of diseases marked by cell death dysregulation. By providing detailed insights into the mechanisms of cell death in these physiologically relevant 3D models, our approach paves the way for more accurate disease modeling and the potential development of targeted therapies^14^.

In conclusion, the RIP3-caspase3-assay represents a significant advancement in organoid research. The application of this assay to spheroid cultures, a first in this field, has demonstrated its robustness and reliability in discerning between various cell death mechanisms, such as apoptosis and necroptosis.

### Supplementary Information

Supplementary Information.

## Data Availability

The datasets used and/or analyzed during the current study are available from the corresponding author on request.

## Abbreviations

AdSC: Adult stem cells
FACS: Fluorescence activated cell sorting
FSC-A: Forward scatter area
IL-1ꞵ: Interleukin 1 beta
IL-6: Interleukin 6
NF-κB: Nuclear factor kappa-light-chain-enhancer of activated B-cells
PI: Propidium iodide
TNFα: Tumor necrosis factor-alpha
RIP1: Receptor-interacting serine-threonine protein kinase 1
RIP3: Receptor-interacting serine-threonine protein kinase 3
ROCK: Rho-kinase
SSC-A: Side scatter area
