# Supplementary material for: Exploring cell death mechanisms in spheroid cultures using a novel application of the RIP3-caspase3-assay
Source: Sci Rep. 2024 Jul 11;14:16032. doi: 10.1038/s41598-024-66805-4 (PMC11239891; doi:10.1038/s41598-024-66805-4)
Supplement: Supplementary file 1 — Supplementary Information. [file 41598_2024_66805_MOESM1_ESM.docx]

**Supplementary Material**

| **patient** | **sex** | **age at operation** | **diagnosis** |
| --- | --- | --- | --- |
| **Nr. 1** | male | 3.5 months | Hirschsprung’s Disease |
| **Nr. 2** | female | 6.0 months | Anorectal Malformation |
| **Nr. 3** | male | 10.5 months | Hirschsprung’s Disease |
| **Nr. 4** | female | 9.0 months | Anorectal Malformation |
| **Nr. 5** | male | 3.0 months | Hirschsprung’s Disease |

***Tab. 2s****: Patient characteristics from organoid original samples.*


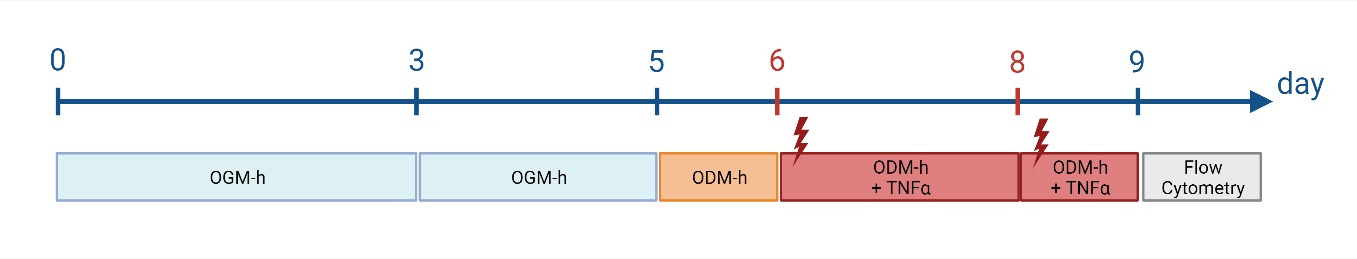


**Fig.3s**: Scheme of the medium changes and the addition of TNFα during the 9 days of experiment for determination of cytokine concentration. Growth medium (blue) and differentiation medium (orange) were changed on the indicated days. TNFα (red) was added on day 6 and 8 to induce proinflammatory stress. Figure created with BioRender.com.


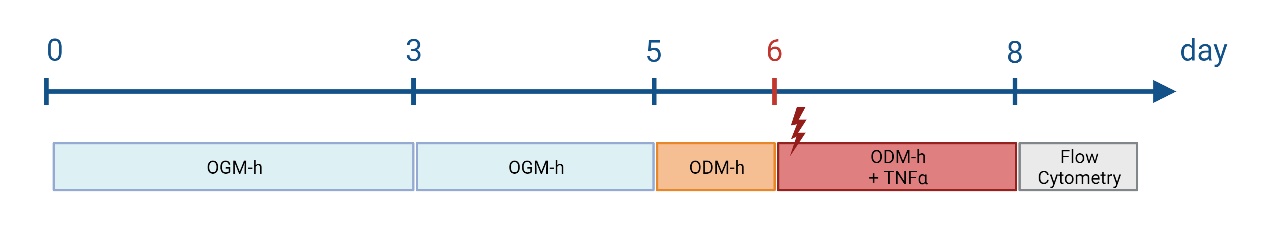


***Fig. 4s****: Scheme of the medium changes and the addition of TNFα during the 8 days of RIP3-Caspase3 assay experiment. Growth medium (blue) and differentiation medium (orange) were changed on the indicated days. TNFα (red) was added on day 6 to induce proinflammatory stress. Figure created with BioRender.com.*

**A**

**B**


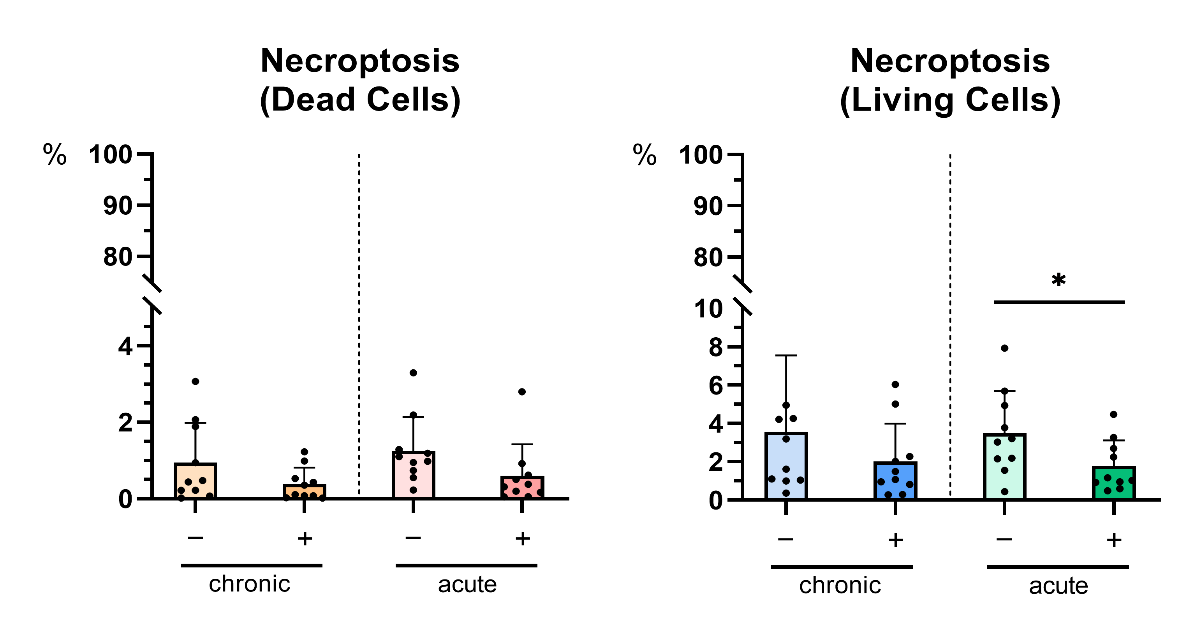


***Fig. 5s:*** *Increased necroptosis detection rates after stimulation of intestinal colonic organoids (n=10) with a proinflammatory cytokine cocktail containing TNFα, IL-6 and IL-1ꞵ. Stimulation was carried out in either an acute (days 5, 6 and 7) or chronic (days 3 and 5) manner. FACS was performed after day 7 and 8, respectively.* **A** *Zombie^+^ dead cells show a reduction in RIP3^+^/Caspase3^-^ cells after proinflammatory stimulation.* **B** *Living cells (Zombie^-^) also showed a decrease in necroptosis following treatment, with reaching significance for acute samples (p=0.0371). Reduction may be explained by a shift of cell death mechanisms towards apoptosis following stimulation (not shown), since cell death mechanisms are displayed as parts of 100. (Li and Hagens et al., unpublished data)*
